# Effects of Acute Organophosphorus Poisoning on Function of Peripheral Nerves: A Cohort Study

**DOI:** 10.1371/journal.pone.0049405

**Published:** 2012-11-20

**Authors:** Sudheera S. Jayasinghe, Kithsiri D. Pathirana, Nick A. Buckley

**Affiliations:** 1 Department of Pharmacology, Faculty of Medicine, University of Ruhuna, Galle, Sri Lanka; 2 South Asian Clinical Toxicology Research Collaboration, Faculty of Medicine, University of Peradeniya, Peradeniya, Sri Lanka; 3 Department of Medicine, Faculty of Medicine, University of Ruhuna, Galle, Sri Lanka; 4 Prince of Wales Hospital Clinical School, University of NSW, Sydney, Australia; University of Chicago, United States of America

## Abstract

**Background:**

Following acute organophosphorus (OP) poisoning patients complain of numbness without objective sensory abnormalities or other features of OP induced delayed polyneuropathy. The aim of this study was to measure peripheral nerve function after acute exposure to OP.

**Methods:**

A cohort study was conducted with age, gender and occupation matched controls. Motor nerve conduction velocity (MNCV), amplitude and area of compound muscle action potential (CMAP), sensory nerve conduction velocity (SNCV), F- waves and electromyography (EMG) on the deltoid and the first dorsal interosseous muscles on the dominant side were performed, following acute OP poisoning. All neurophysiological assessments except EMG were performed on the controls.

Assessments were performed on the day of discharge from the hospital (the first assessment) and six weeks (the second assessment) after the exposure. The controls were assessed only once.

**Results:**

There were 70 patients (50 males) and 70 controls. Fifty-three patients attended for the second assessment.

In the first assessment MNCV of all the motor nerves examined, CMAP amplitude and SNCV of ulnar nerve, median and ulnar F-wave occurrence in the patients were significantly reduced compared to the controls.

In the second assessment significant reduction was found in SNCV of both sensory nerves examined, MNCV of ulnar nerve, CMAP amplitude of common peroneal nerve, F-wave occurrence of median and ulnar nerves.

No abnormalities were detected in the patients when compared to the standard cut-off values of nerve conduction studies except F-wave occurrence.

EMG studies did not show any abnormality.

**Conclusion:**

There was no strong evidence of irreversible peripheral nerve damage following acute OP poisoning, however further studies are required.

## Introduction

Organophosphate (OP) compounds have become the most widely used pesticides for agricultural-pests throughout the world from the 1980s and the risk of acute and sub acute toxicity is high in humans [1]. Acute pesticide poisoning is a major health problem especially in developing countries. It was estimated that one million serious, unintentional poisonings occurred and an additional two million people were hospitalized for attempted suicide with pesticides annually [2]. In Sri Lanka, the majority of poisoning cases are self inflicted and 77% of the cases are in the age range of 11–30 years [3]. OP and carbamate compounds were involved in 74% of pesticide poisoning [3], [4].

OP poisoning leads to four well defined neurological syndromes, namely acute cholinergic crisis, intermediate syndrome, organophosphate induced delayed polyneuropathy (OPIDN) and chronic organophosphate induced neuropsychiatric disorders (COPIND) [5].

Animal studies have shown axonal degeneration and demyelination following acute OP poisoning [6]. Chronic neuropsychological dysfunction with a single episode of acute unintentional OP intoxication has also been reported [7]. Human studies of nerve function in farm workers who had chronic, probably low level exposure to pesticides show sensory and motor neuropathy [8], [9], [10], [11], [12]. Systematic studies which focus on peripheral nerve function with acute OP ingestion are scant. The aim of the study was to find out whether there is any evidence of sub-clinical axonal damage and demyelination following acute OP poisoning in humans.

## Materials and Methods

A cohort study was conducted with matched controls with the approval of the Ethical Review Committee, Faculty of Medicine, University of Ruhuna, Sri Lanka. Informed written consent was obtained from the patients and the controls. All clinical investigations were conducted according to the principles expressed in the Declaration of Helsinki.

The patients with self ingestion of OP were recruited from a tertiary care hospital and a secondary care hospital in the Southern Province of Sri Lanka between June 2008 and September 2009. At the time of recruitment to the study, subjects either had features of the cholinergic syndrome or had been given atropine to counteract cholinergic syndrome in the peripheral units and then transferred to the collaborating hospitals.

OP poisoning was confirmed by the history from the patient and/or accompanying person, the cholinergic features and plasma cholinesterase activity (ChE).

The controls were recruited from the persons accompanying the patients to the tertiary care hospital. Age, gender and occupation matched healthy volunteers who did not have a history of acute pesticide exposure were recruited within one month of the recruitment of the respective case. Age of the controls was matched to ±3 years of the patients.

Subjects with features of peripheral neuropathy, diabetes mellitus or those who were on long term medications, were excluded.

Motor nerve conduction studies (MNCS), sensory nerve conduction studies (SNCS), F-wave studies and electromyography (EMG) were performed at the time of discharge from the ward (the first assessment) and at six weeks (the second assessment) following acute exposure to OP. All neurophysiological investigations done on the patients were carried out on the controls except EMG. The room temperature of the neurophysiology laboratory was maintained at 25°C.

The patients were assessed twice to explore the acute and subsequent effects on peripheral nerves. The earliest possible time to assess the cases was at the time of discharge.

Immediately after the development of an acute neuropathic lesion, EMG does not show any abnormalities. The subsequent changes depend on the occurrence of denervation. The appearance of denervation on EMG may be delayed for up to five weeks [13]. Therefore the earliest possible time for the second assessment with the least drop outs was at the sixth week.

The Neuropack MEB-9400A/K EMG/EP Measuring System (Nihon Koden) was used for electrophysiological assessment.

### SNCS

SNCS were performed on median and ulnar nerves on both sides. The orthodromic method was used. Stimulating ring electrodes were placed on the second digit for median SNCS and on the fourth digit for ulnar SNCS. The ring cathode was placed around the digit near the metacarpophalangeal joint and the ring anode around the digit near the distal interphalangeal joint. A recording electrode was placed over the respective nerve on the anterior aspect of the wrist. Ground electrode was placed between the stimulating and the recording electrodes. The nerve was stimulated with supramaximal electrical stimulus.

Sensory nerve conduction velocities (SNCV) and the amplitude of the complex were recorded.

### MNCS

MNCS were performed on median, ulnar and common peroneal nerves on both sides. Surface electrodes were used. The nerve was stimulated with supramaximal stimulation.

For the median MNCS, the recording electrode was placed over the abductor pollicis brevis, the reference electrode was placed over the proximal phalanx of the thumb and the ground electrode was attached between recording electrode and the stimulating probe. The stimulation was given on the palmar aspect of midwrist and at the elbow (antecubital fossa) just medial to the palpable brachial artery. The cathode was placed distal to the anode.

For the ulnar MNCS the recording electrode was placed on the mid portion of the abductor digiti quinti, the reference electrode was placed over the proximal phalanx of the fifth digit and the ground electrode was attached between recording electrode and the stimulating probe. The nerve was stimulated on the palmar aspect of wrist and just distal to the osseous groove in the posterior aspect of the medial epicondyle of the humerus, i.e, the ulnar groove.

For the common peroneal MNCS the recording electrode was placed over the extensor digitorum brevis muscle, the reference electrode was placed over the fifth toe and the ground electrode between the recording electrode and the stimulating probe. The nerve was stimulated on the lateral aspect of the popliteal fossa just medial to the insertion of the tendon of the biceps femoris and along the anterolateral surface of the fibula, 3 to 4 cm distal to the proximal tip of the fibular head.

Motor nerve conduction velocity (MNCV), amplitude and area of the compound muscle action potential (CMAP) on distal stimulation were recorded.

### F-wave studies

F wave studies were performed on median, ulnar and tibial nerves. Electrodes were placed as for MNCS on median and ulnar nerves. For the tibial F-wave studies, the recording, the reference and the ground electrodes were placed over the abductor hallucis muscle between the great toe and between the recording electrode and the stimulating probe respectively. The nerve was stimulated at a point slightly posterior and proximal to the medial malleolus. Sixteen stimulations were analyzed, percentage of F-wave occurrence and minimum reproducible F-wave latency were recorded.

### EMG studies

EMG studies were performed on the deltoid and the first dorsal interosseous muscle on the dominant side. The ground electrode was placed on the same side of the upper limb. A concentric needle electrode was inserted into each of the selected muscles at rest and during contraction. Spontaneous activity at rest, amplitude of the motor units, presence or absence of polyphasia and the interference pattern during muscle contraction were noted.

### Estimation of plasma ChE activity

Generally the term acetylcholinesterase activity is referred to red blood cell acetylcholinesterase or acetylcholinesterase at the nerve tissue. However we analyzed ChE (ChE = acetylcholinesterase (AChE) plus butyrylcholinesterase (BChE)) activity in plasma. The modified Ellman method developed by Worek F et al. (1999) was used to estimate ChE activity in plasma [14]. Plasma samples were obtained from EDTA blood after centrifugation (10 min, 500 * g) and stored in 1 ml aliquots at −80°C until analysis.

Prior to analysis the thawed samples were kept on ice until analysis. Preparation of inhibited cholinesterase were made by incubating plasma samples with PX-ethyl, PX-methyl and obidoxime for 15 min at 37°C followed by immediate dilution of the samples (1∶100 in diluting reagent) and freezing.

The activity of cholinesterase was measured with a thermostatted filter photometer (Model 1101 M, Eppendorf, Hamburg, Germany) at 436 nm and 37°C using polystyrol cuvets.

Cholinesterase enzyme activity was calculated by using the equation,
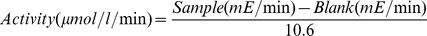
The analysis was done at Walther Straub Institute for Pharmacology and Toxicology, Munich, Germany.

### Statistical analysis

Graph Pad Prism 4 and Social Package of Statistical Software were used for the statistical analysis. Normal distribution of the data was tested with Kolmogorov-Smirnov test. Data which had no normal distribution were analyzed with non-parametric tests. The paired T-test was used to compare the results of the first and the second assessment and the unpaired T-test was used to compare the results of the patients and the controls. Correlation of neurophysiological parameters with potential confounders (alcohol consumption; regular, occasional or none, smoking habits; regular, occasional, none, type of OP ingested, minimum plasma ChE activity, pralidoxime (PAM) therapy and Glasgow Coma Scale (GCS) on admission) were evaluated using multiple linear regression model.

## Results

**Figure 1:**
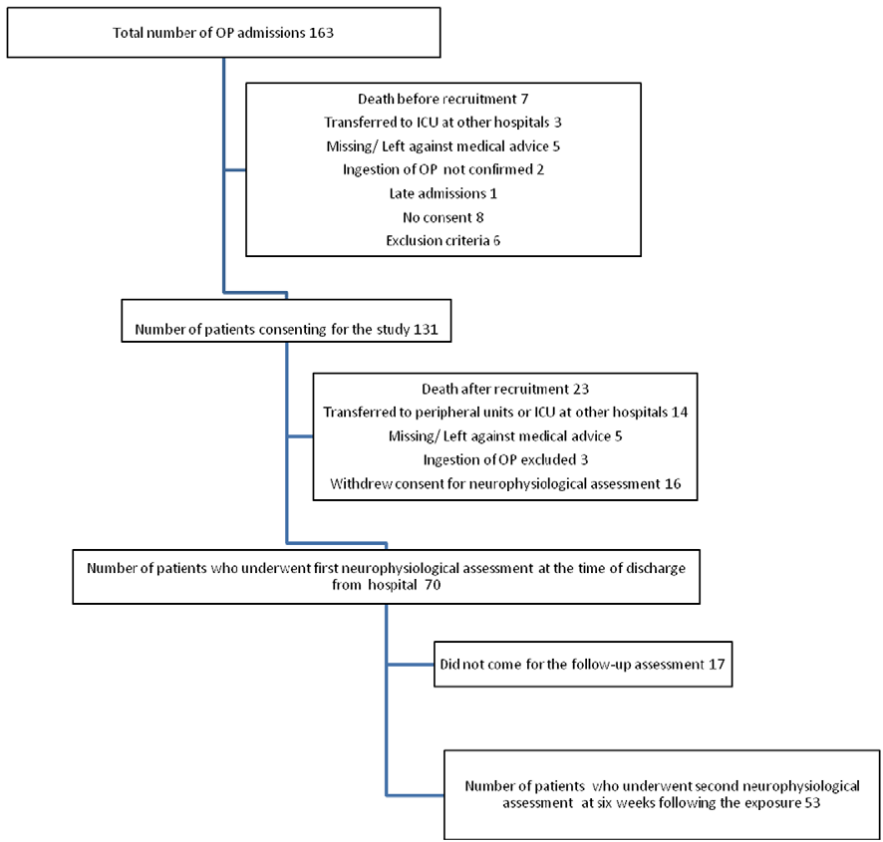
Flow diagram of recruitment of participants.

From a total of 163 acute OP poisoning admissions to the collaborating hospitals, 70 (50 males) patients underwent the first electrophysiological assessment in median (inter quartile range) of 6 (4–7) days following the exposure. Fifty three came for the second assessment at six weeks following the exposure (Figure 1). Mean (SD) GCS on admission was 14 (2). All patients received atropine, 54 patients received pralidoxime.

Plasma ChE activity at four and/or twelve hours after the exposure was available in 33 patients. The median (inter quartile range) of plasma ChE activity at four and twelve hours was 790 (146–2598) µmol/l/min and 431 (136–3068) µmol/l/min respectively.

None of the patients or the controls had diabetis mellitus. The mean HbA_1_C of patients and the controls were 5.4±0.5% and 5.7±0.6%. Descriptive data of the patients and the controls are shown in the Table 1.

**Table 1:** Descriptive data of the patients and the controls.

Descriptive data	Number of patients (n = 70)	Number of controls (n = 70)
Age (years)Φ	31.8 (12.2)	32.7 (11.9)
Height (cm)Φ	158.7 (7.5)	158.7 (10.6)
Weight (kg)Φ	54.9 (10.8)	54.7 (9.4)
*Alcohol consumption*		
No	36	41
Yes - occasional	18	5
Yes - regular	16	24
*Smoking*		
No	40	43
Yes - occasional	5	2
Yes - regular	25	25

Φ Values are mean (SD).

Table 2 presents the numbers of subjects exposed to individual specific OPs.

**Table 2:** Number of poisoned cases by type of OP.

Type of OP	Number of poisoned cases
Chlorpyrifos	26
Dimethoate	12
Profenofos	5
Diazinon	4
Malathion	2
Fenthion	1
Others	3
Type of OP was not identified	17

**Figure 2:**
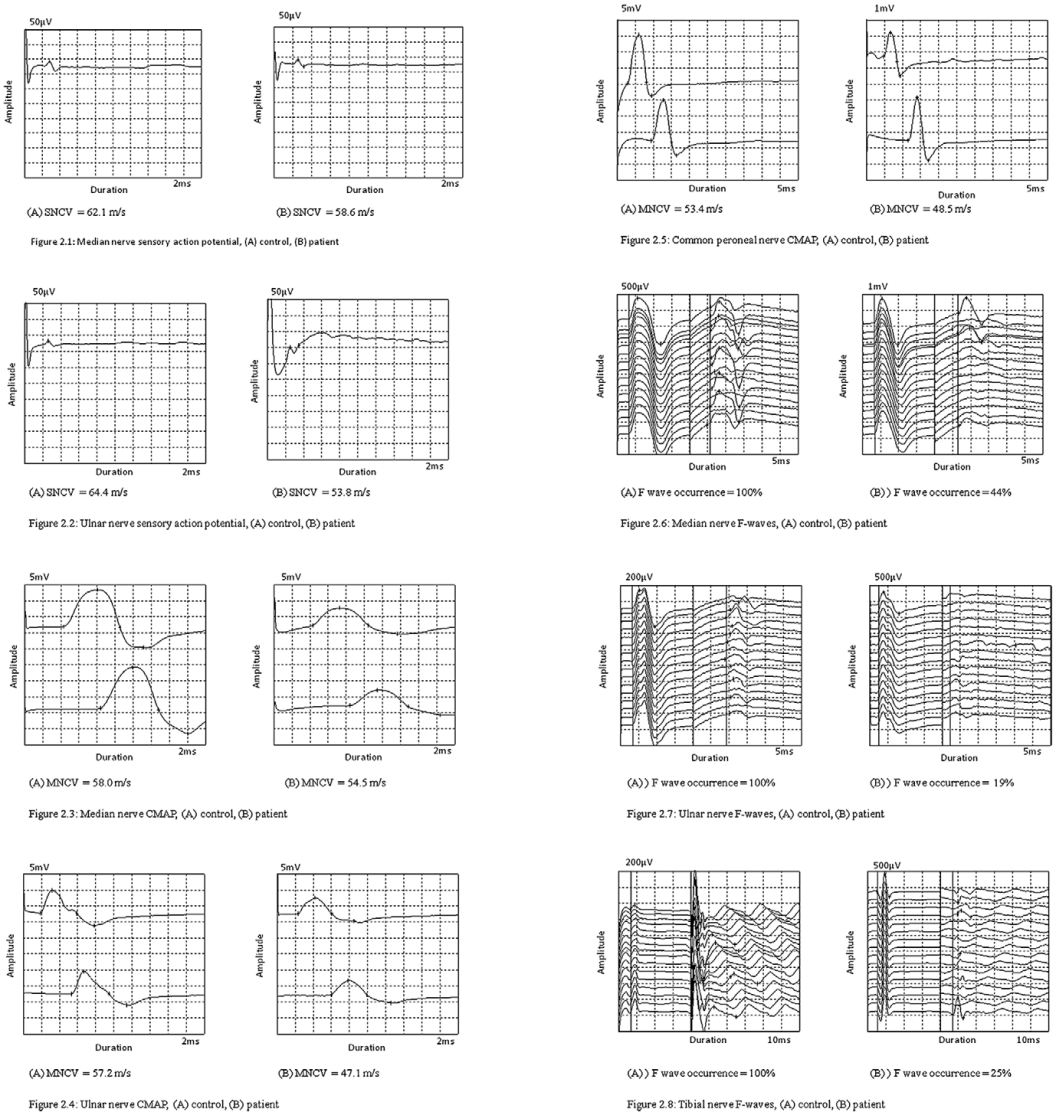
Wave forms of nerve conduction studies. Figure 2.1 - Median nerve sensory action potential, (A) control, (B) patient. Figure 2.2 - Ulnar nerve sensory action potential, (A) control, (B) patient. Figure 2.3 - Median nerve CMAP, (A) control, (B) patient. Figure 2.4 - Ulnar nerve CMAP, (A) control, (B) patient. Figure 2.5 - Common peroneal nerve CMAP, (A) control, (B) patient. Figure 2.6 - Median nerve F-waves, (A) control, (B) patient. Figure 2.7 - Ulnar nerve F-waves, (A) control, (B) patient. Figure 2.8 - Tibial nerve F-waves, (A) control, (B) patient.

The number of participants who underwent individual neurophysiological assessment (Table 3) and the results of SNCS, MNCS and F-wave studies are shown in the Table 4. The wave forms of nerve conduction studies are shown in the Figure 2. Impairment of peripheral nerve function was observed at both occasions in the cases compared to the controls. In the first assessment these were significant for MNCV of median, ulnar and common peroneal nerves, SNCV and CMAP amplitude of ulnar nerve and F wave occurrence of median, ulnar and tibial nerves. However no abnormality was detected when compared to the standard cut-off values for normal MNCS and SNCS except F-wave occurrence. In the second assessment significant worsening of peripheral nerve function was seen in common peroneal CMAP-area and reduction of tibial F-wave latency compared to the first assessment. MNCV of median and common peroneal nerves, amplitude of ulnar nerve CMAP on distal stimulation and F-wave occurrence of tibial nerve reversed to normal at the second assessment. EMG was performed in 42 patients who had given consent in the first assessment and 31 patients in the second assessment. None of the patients showed fibrillation potentials or evidence of denervation (high amplitude, polyphasia and reduced recruitement of motor units).

**Table 3:** The number of participants who underwent individual neurophysiological assessment.

Neurophysiological assessment	First assessment of the patients (n = 70)	Second assessment of 2the patients (n = 53)	Controls (n = 70)
Median sensory nerve conduction studies	61	48	68
Ulnar sensory nerve conduction studies	58	45	70
Median motor nerve conduction studies	70	53	69
Ulnar motor nerve conduction studies	70	53	70
Common peroneal motor nerve conduction studies	66	48	68
Median F-wave studies	70	53	69
Ulnar F-wave studies	69	53	69
Tibial F-wave studies	65	50	65

**Table 4:** Effects of acute OP exposure on peripheral nerve function compared to healthy controls.

Neurophysiological assessment	First assessment of the patients	Second assessment of the patients	Controls	Difference (Controls - 1^st^ assessment)	95% CI/P value (Controls - 1^st^ assessment)	Difference (Controls - 2^nd^ assessment)	95% CI/P value (Controls - 2^nd^ assessment)	Difference (1^st^ assessment - 2^nd^ assessment)	95% CI/P value (1^st^ assessment - 2^nd^ assessment)
***SNCV (m/s)***									
Median	53.6 (1.0)	52.8 (1.2)	56.0 (0.7)	2.3	−0.2 to 4.7	3.2¥	0.6 to 5.8	1.2	−0.6 to 3
Ulnar	55.3 (0.9)	55.9 (1.0)	59.7 (0.6)	4.4¥	2.3 to 6.5	3.7¥	1.5 to 5.9	0.01	−2.5 to 2.5
***Amplitude of sensory complex (µV)***									
Median	14.3 (10.4)	12.9 (10.6)	13.4 (7.1)	−0.9	−4 to 2	0.5	−3 to 4	0.5	−5 to 4
Ulnar	7.4 (6.3)	7.2 (7.4)	7.7 (4.3)	0.3	−2 to 2	0.4	−2 to 3	0.8	−4 to 3
***MNCV (m/s)***									
Median	55.2 (0.5)	55.6 (0.6)	56.6 (0.4)	1.4¥	0.1 to 2.7	1.05	−0.4 to 2.5	−0.8	−1.7 to 0.06
Ulnar	53.9 (0.6)	54.4 (0.7)	56.2 (0.5)	2.3¥	0.7 to 3.8	1.8¥	0.1 to 3.5	−0.4	−1.4 to 0.7
Common peroneal	46.6 (0.6)	48.2 (0.7)	49.4 (0.6)	2.8¥	1.1 to 4.5	1.2	−0.7 to 3.1	−1.2	−2.3 to 0.1
***Amplitude of CMAP on distal stimulation (mV)***									
Median	13.4 (0.4)	14.2 (0.6)	14.4 (0.5)	0.9	−0.4 to 2.3	0.1	−1.5 to 1.7	−0.7	−1.5 to 0.08
Ulnar	9.5 (0.3)	10.0 (0.3)	10.5 (0.3)	1.0¥	0.2 to 1.8	0.5	−0.4 to 1.3	−0.3	−0.8 to 0.3
Common peroneal	7.7 (0.4)	7.2 (0.4)	8.7 (0.4)	0.9	−0.2 to 2.1	1.4¥	0.3 to 2.6	0.6	−0.2 to 1.3
***Area of CMAP on distal stimulation (mVms)***									
Median	32.3 (1.2)	30.9 (1.4)	33.6 (1.2)	1.4	−2.0 to 4.8	2.7	−0.9 to 6.3	0.9	−0.7 to 2.7
Ulnar	18.7 (0.8)	17.1 (0.7)	19.5 (0.5)	0.8	−1.0 to 2.6	2.3¥	0.6 to 4	1.3	−0.02 to 2.6
Common peroneal	15.3 (0.8)	13.1 (0.7)	16.2 (0.8)	0.9	−1.4 to 3.2	3¥	0.8 to 5.2	2.7¥	1.2 to 4.2
***F-wave latency (ms)***									
Median	27.2 (0.4)	26.8 (0.3)	26.7 (0.3)	−0.4	−1.3 to 0.4	−0.1	−0.9 to 0.7	0.3	−0.4 to 0.9
Ulnar	27.0 (0.3)	27.3 (0.4)	26.7 (0.3)	−0.3	−1.2 to 0.5	−0.6	−1.6 to 0.3	−0.3	−0.9 to 0.3
Tibial	50.9 (0.6)	49.4 (0.8)	49.6 (0.6)	−1.3	−3.1 to 0.4	0.2	−1.7 to 2.1	1.9¥	0.2 to 3.6
***F-wave occurrence (%)***									
Median	82 (2)	78 (3)	90(1)	9¥	0.005†	12¥	0.002†	3	0.4‡
Ulnar	83(3)	84(2)	93 (1)	10¥	0.001†	9¥	0.002†	−2	0.6‡
Tibial	89(2)	92 (2)	93 (2)	4	0.059†	1	0.6†	−0.3	0.8‡

values are as mean and SE, analyzed using.

† Mann-Whitney U test and

‡ Wilcoxon signed rank test,

¥ significant at 0.05 level.

Seventeen patients were admitted to Intensive Care Unit. Among them seven patients died. Four of the 10 (40%) survivors underwent neurophysiological assessment at median (range) of 24 (6–40) days after the exposure.

Correlation of neurophysiological indices with potential confounders is shown in the Table 5. None of the neurophysiological indices significantly correlate with plasma ChE activity. However MNCV of ulnar; SNCV of median and ulnar, MNCV of ulnar; MNCV of median, ulnar and tibial F-wave occurrence showed significant negative Spearman's correlations with smoking habits, PAM therapy and alcohol consumption respectively.

**Table 5:** Correlation matrix of neurophysiological indices with potential confounders. The analysis was done with Spearman's correlation.

Neurophysiological assessment	Alcohol consumption	Smoking habits	ChE activity	Type of OP ingested	GCS on admission	PAM therapy
***SNCV (m/s)***						
Median	−0.004 (0.8)	−0.03 (0.8)	0.09 (0.6)	−0.06 (0.7)	0.1 (0.4)	−0.3 (0.03)¥
Ulnar	−0.006 (0.6)	−0.09 (0.5)	−0.01 (0.9)	−0.2 (0.06)	0.09 (0.5)	−0.3 (0.04)¥
***MNCV (m/s)***						
Median	−0.2 (0.4)	−0.2 (0.04)¥	0.02 (0.9)	−0.06 (0.6)	−0.1 (0.4)	−0.2 (0.09)
Ulnar	−0.3 (0.005)¥	−0.3 (0.008)¥	−0.09 (0.6)	−0.09 (0.5)	0.02 (0.8)	−0.3 (0.009)¥
Common peroneal	−0.2 (0.1)	−0.2 (0.05)¥	−0.1 (0.5)	−0.01 (0.9)	0.2 (0.1)	0.03 (0.8)
***Amplitude of CMAP on distal stimulation (mV)***						
Median	0.03 (0.8)	−0.1 (0.3)	−0.4 (0.5)	0.2 (0.09)	0.3 (0.01)¥	−0.2 (0.06)
Ulnar	−0.1 (0.3)	−0.008 (0.5)	−0.2 (0.4)	−0.1 (0.2)	0.2 (0.07)	−0.1 (0.4)
Common peroneal	−0.1 (0.3)	−0.1 (0.2)	0.2 (0.2)	0.01 (0.9)	0.04 (0.7)	−0.04 (0.7)
***Area of CMAP on distal stimulation (mVms)***						
Median	0.07 (0.5)	−0.06 (0.6)	−0.3 (0.1)	0.09 (0.4)	0.2 (0.04)¥	0.3 (0.01)¥
Ulnar	0.1 (0.4)	0.1 (0.3)	−0.2 (0.4)	−0.3 (0.009)¥	0.1 (0.2)	−0.1 (0.4)
Common peroneal	−0.1 (0.4)	−0.1 (0.3)	0.2 (0.3)	<0.001 (0.9)	0.09 (0.5)	−0.1 (0.4)
***F-wave latency (ms)***						
Median	0.3 (0.02)¥	0.2 (0.05)¥	0.2 (0.2)	0.05 (0.7)	0.02 (0.9)	0.005 (0.9)
Ulnar	0.3 (0.02)¥	0.4 (0.003)¥	0.2 (0.2)	0.08 (0.5)	−0.004 (0.9)	0.03 (0.8)
Tibial	0.2 (0.06)	0.3 (0.04)¥	0.1 (0.5)	0.09 (0.4)	0.01 (0.9)	−0.2 (0.1)
***F-wave occurrence (%)***						
Median	−0.02 (0.9)	−0.1 (0.3)	−0.2 (0.2)	0.1 (0.3)	0.1 (0.3)	−0.05 (0.7)
Ulnar	0.1 (0.4)	0.004 (0.9)	−0.2 (0.3)	0.1 (0.2)	−0.008 (0.9)	−0.01 (0.4)
Tibial	0.1 (0.4)	0.1 (0.4)	−0.1 (0.5)	0.1 (0.4)	0.03 (0.8)	−0.3 (0.02)¥

Values are Spearman's correlation coefficient (P value),

¥ Significant at 0.05 level.

When multiple liner regression models were used to adjust for potential confounders, effects on SNCV of median nerve, area of CMAP of median nerve and F-wave latency of tibial nerve with PAM therapy and the area of CMAP of ulnar nerve with the type of OP ingested showed statistical significance (Table 6 and 7).

**Table 6:** Sensory and motor nerve conduction studies - multiple linear regression for confounders.

Dependent variable	Independent variables	Regression (B)	Standard error of B (SE)	95% confidence interval for β
***SNCV (m/s)***				
**Median**	Alcohol consumption	−0.8	1.8	−4 to 3
	Smoking habits	1.0	1.6	−2 to 4
	Type of OP	−0.4	1.5	−4 to 3
	PAM therapy	−5.9	2.7	−11 to −0.7¥
	GCS on admission	0.4	0.4	−0.4 to 1.2
**Ulnar**	Alcohol consumption	−0.6	1.6	−3 to 3
	Smoking habits	−0.3	1.4	−3 to 2
	Type of OP	−2.7	1.4	−5 to 0.05
	PAM therapy	−3.4	2.3	−8 to 1
	GCS on admission	0.03	0.4	−0.7 to 0.8
***MNCV (m/s)***				
**Median**	Alcohol consumption	−1.0	0.9	−3 to 0.7
	Smoking habits	−0.3	0.8	−2 to 1.3
	Type of OP	−0.4	0.7	−2 to 1
	PAM therapy	−1.2	1.3	−4 to 1.4
	GCS on admission	−0.1	0.2	−0.6 to 0.3
**Ulnar**	Alcohol consumption	−1.5	0.5	−3 to 0.4
	Smoking habits	−0.7	0.5	−2 to 0.9
	Type of OP	−1.1	0.4	−3 to 0.5
	PAM therapy	−1.8	0.8	−5 to 1
	GCS on admission	−0.07	0.1	−0.5 to 0.4
**Common peroneal**	Alcohol consumption	−0.5	0.7	−3 to 2
	Smoking habits	−0.9	0.6	−3 to 1
	Type of OP	−0.3	0.6	−2 to 1.5
	PAM therapy	0.8	1.1	−2 to 4
	GCS on admission	0.1	0.2	−0.4 to 0.7
***Amplitude of CMAP on distal stimulation (mV)***				
**Median**	Alcohol consumption	1.1	0.7	−0.3 to 2.5
	Smoking habits	−0.8	0.6	−2 to 0.5
	Type of OP	1.1	0.6	−0.09 to 2
	PAM therapy	−2.1	1.1	−4 to 0.04
	GCS on admission	0.2	0.2	−0.2 to 0.5
**Ulnar**	Alcohol consumption	−0.4	0.5	−1.4 to 0.6
	Smoking habits	−0.1	0.5	−1 to 0.8
	Type of OP	−0.7	0.4	−1.5 to 0.2
	PAM therapy	0.08	0.8	−1.5 to 1.6
	GCS on admission	0.2	0.1	−0.03 to 0.9
**Common peroneal**	Alcohol consumption	−0.3	0.7	−2 to 1
	Smoking habits	−0.4	0.6	−2 to 0.8
	Type of OP	−0.4	0.6	−2 to 0.8
	PAM therapy	0.01	1.1	−2 to 2
	GCS on admission	0.1	0.2	−0.2 to 0.5
***Area of CMAP on distal stimulation (mVms)***				
**Median**	Alcohol consumption	3.1	2.1	−1 to 7
	Smoking habits	−1.9	1.8	−6 to 2
	Type of OP	1.3	1.7	−2 to 5
	PAM therapy	−7.3	3.1	−14 to −1¥
	GCS on admission	0.5	0.5	−0.5 to 2
**Ulnar**	Alcohol consumption	−0.6	1.3	−3 to 2
	Smoking habits	1.0	1.1	−1 to 3
	Type of OP	−3.0	1.0	−5 to −0.9¥
	PAM therapy	−1.3	1.9	−5 to 3
	GCS on admission	0.5	0.3	−0.1 to 1.1
**Common peroneal**	Alcohol consumption	−0.7	1.4	−4 to 2
	Smoking habits	−0.6	1.3	−3 to 2
	Type of OP	−0.6	1.2	−3 to 2
	PAM therapy	−0.9	2.2	−5 to 3
	GCS on admission	0.3	0.4	−0.5 to 0.9

¥ Significant at 0.05 level.

**Table 7:** F wave studies - multiple linear regression for confounders.

Dependent variable	Independent variables	Regression (B)	Standard error of B (SE)	95% confidence interval for β
***F-wave latency (ms)***				
**Median**	Alcohol consumption	0.6	0.6	−0.6 to 1.8
	Smoking habits	0.5	0.5	−0.5 to 1.6
	Type of OP	0.3	0.5	−0.7 to 1.2
	PAM therapy	−0.1	0.9	−2 to 2
	GCS on admission	0.1	0.1	−0.2 to 0.4
**Ulnar**	Alcohol consumption	1.1	0.6	−0.08 to 2
	Smoking habits	0.2	0.5	−0.8 to 1.2
	Type of OP	0.8	0.5	−0.2 to 1.7
	PAM therapy	0.1	0.8	−1.6 to 1.8
	GCS on admission	0.2	0.1	−0.1 to 0.4
**Tibial**	Alcohol consumption	0.8	1.1	−1.3 to 3
	Smoking habits	1.3	0.9	−0.5 to 3
	Type of OP	0.7	0.8	−0.9 to 2.4
	PAM therapy	−3.6	1.5	−7 to −0.7¥
	GCS on admission	0.2	0.2	−0.2 to 0.7
***F-wave occurrence (%)***				
**Median**	Alcohol consumption	4.5	3.6	−3 to 12
	Smoking habits	−3.2	3.1	−10 to 3
	Type of OP	3.1	2.9	−3 to 9
	PAM therapy	−1.2	5.4	−11 to 10
	GCS on admission	−0.9	0.9	−3 to 0.9
**Ulnar**	Alcohol consumption	5.7	4.3	−3 to 14
	Smoking habits	−1.4	3.8	−9 to 6
	Type of OP	5.7	3.6	−2 to 13
	PAM therapy	−2.3	6.5	−15 to 11
	GCS on admission	0.2	1.1	−2 to 2
**Tibial**	Alcohol consumption	−2.1	3.5	−9 to 5
	Smoking habits	4.3	3.0	−2 to 10
	Type of OP	2.5	2.8	−3 to 8
	PAM therapy	−11.9	4.9	−22 to 2
	GCS on admission	1.2	0.8	−0.4 to 3

¥ Significant at 0.05 level.

To determine whether any multicollinearity were present, and to understand whether there is a strong linear association between each predictor variable and all other remaining predictors, the Variance Inflation Factor (VIF) and condition indices were examined. None of the VIF exceeds 10 and a condition index exceeds 30.

## Discussion

We observed small magnitude adverse difference of SNCV, MNCV, amplitude and area of CMAP on distal stimulation and F-wave occurrence in acute OP poisoned patients compared to the controls.

ChE activity in patients poisoned with dimethoate may be high. In contrast the active metabolite of chlorpyrifos (chlorpyrifos-oxon) is more potent and inhibits BChE more than AChE [15]. In chlorpyrifos poisoning, all patients with sufficient AChE inhibition to provide clinical symptoms will have markedly decreased BChE activity [15]. Among 26 patients identified as chlorpyrifos ingestion from the label of the containers brought to the hospital, six patients showed high levels of ChE. All six patients had cholinergic features on admission and treated with atropine and pralidoxime. High levels of ChE activity may be due to incorrect identification of poison or mixed ingestion with dimethoate. It was unlikely to be due to mild ingestion since patients had full blown cholinergic syndrome at the time of admission. Over all high levels of ChE of our patients may be due to mild toxic patients and dimethoate poisoned patients.

Diabetes is well known to cause neuropathy. Therefore we excluded patients with diabetes from the study. Occupation matched controls were recruited since there is evidence that occupational exposure to pesticides can cause neuropathies. Very few studies were found in the literature which focused on SNCS/MNCS following acute ingestion of OP (Table 8). There have been no studies which have looked at the effects of acute OP exposure on peripheral nerve function compared to matched controls. Most large studies have examined nerve function in farm workers who had chronic, probably low level exposure to pesticides (Table 8). Reduction of SNCV and MNCV of the patients in our study indicate that there may be demyelination following acute OP exposure. Since we did not follow up the patients beyond six weeks of exposure we do not know whether the changes were reversed or worsened with time.

**Table 8:** Studies of pesticide exposure and peripheral nerve function.

Study	Exposure state	Exposed population	Substance	Number	Comparison group	Findings
Boildin et al (1979) [6]	Acute	Adult cats	DFP (OP)	5	none	Focal distal non terminal axonal degeneration
Jayawardana et al (2008) [20]	Acute	Patients	OP	60	none	Normal sensory and motor nerve conduction
Steenland et al (1994) [21]	Acute on chronic	Farm workers	OP	83	Friends	Normal nerve conduction studies (Significant deficit was observed in nerve conduction velocity and/or amplitude among men poisoned by chlorpyrifos or phosalone)
Senanayake et al (1987) [22]	Acute	Patients with intermediate syndrome	OP	6	none	Normal nerve conduction
Senanayake et al (1982) [23]	Acute	Patients	Methamidophos	10	none	EMG denervation, normal nerve conduction
Kamel et al (2004) [8]	Review article	-	Pesticide	-	-	High-level exposure to OP can cause sensory abnormalities and motor dysfunction. But there was less evidence that low to moderate level exposure to OP was related to deficits in sensory or motor function or peripheral nerve conduction.
Steenland et al (2000) [9]	Chronic	Termiticide applicators	Chlorpyrifos	191	friends	Sensory neuropathy
Engle et al (1998) [10]	Chronic	Farm workers	OP	67	Matched reference subjects	Normal nerve conduction studies
Misra et al (1998) [12]	Chronic	Farm workers	Fenthion	24	Same subjects three weeks withdrawal from work	Reduction of peroneal MNCV, distal motor latency of median and peroneal
Ruijten et al (1994) [11]	Chronic	Farm workers	Mixed pesticide	131	Volenteers from the general population	Decreased motor nerve conduction velocity was found in median and peroneal nerves. Sensory nerve conduction velocity was found in median and sural nerves.

Reduced amplitude and/or area of CMAP on distal stimulation were observed in several comparisons (Table 4). These indicate that there may be an axonal damage since amplitude and area under negative curve of CMAP are directly proportional to the number of functioning axons [16]. If the whole length of the nerve is affected, F-wave latency should be prolonged. Reduced nerve conduction velocity only in the distal segment may be an evidence of distal demyelination with sparing of proximal segment. Since we did not perform segmental nerve conduction studies focal damage cannot be excluded.

F-wave studies are also important for recognizing the proximal segment involvement since it is produced by antidromic activation of motor neurons. It is also important to identify marginal changes of standard MNCS since an impulse travels a long pathway to produce an F-wave, and additive effects are more obvious in F-wave latency measurement as it measures the delay in a much longer segment [16]. The latency of an F-wave includes the time required for the evoked action potential to ascend antidromically to the anterior horn cells, the time between the begining of antidromic activation and subsequent orthodromic discharge (the central “turnaround” time) and the time required for the resultant action potential to descend orthodromically from the anterior horn cells to the muscle fibers. F-wave abnormalities may be loss of response (reduction of occurrence) or prolonged latencies [16]. F-waves do not occur with each stimulation. They depend on excitability of motor neurons. In normal individuals F-wave occurrence is more than 90% [16].

In our study there was a statistically significant reduction of F-wave occurrence observed in the median and the ulnar nerves in the first assessment compared to the controls. Although there was prolongation of F-wave latency, it was not statistically significant.

Some nerves such as the ulnar and the common peroneal are highly vulnerable to external damage in the ulnar groove and behind the head of the fibula respectively. Improper nursing care may be partially contributed to the nerve damage.

The clinical usefulness of oxime therapy in OP poisoning is currently not clear. A clinical trial conducted in Sri Lanka with 45 patients did not show any benefit from pralidoxime plus atropine over atropine alone in the management of OP poisoning [17]. A study conducted in Vellore, India showed that low-dose infusion of pralidoxime caused harm [18]. Whereas Pawar K S et al. (2006) showed a high-dose regimen of pralidoxime (constant infusion of 1 g/h for 48 h after 2 g loading dose) reduces the requirement of atropine during the first 24 h, need of intubation and duration of ventilator support than controls who received a bolus dose of 1 g pralidoxime over 1 h every 4 h for 48 h after 2 g of loading dose [19].

### Limitations

Exposure state was not blinded to the researcher since it is not practically possible to blind in this nature of studies. Although long axons of the lower extremities (sural nerve) are considered to be most sensitive for toxic neuropathy, we were unable to evaluate this nerve the current study was not able to investigate it due to technical problem encountered. F-waves are inheritably variable in latency due to varying conduction velocities of individual motor fibers [16]. We have considered F-wave latency as minimum reproducible latency. That does not reflect the individual latency of F-wave series. This may explain why we did not observe any significant difference of F-wave latency. It would have been better if a parameter which could reflect individual latencies of F-wave series have been used (e.g. mean F-wave latency, range of F-wave or dispersion of F-waves) instead of minimum reproducible F-wave latency [16].

### Conclusions

Our study suggests that there may be sub-clinical sensory and motor neuropathies following single acute exposure to OP. Reduced nerve conduction velocity with normal F-wave latency might reflect a distal demyelination process. However the reduced of amplitude of CMAP on distal stimulation may indicates axonal damage. The motor neuron excitability was reduced as reflected by the reduced F-wave occurrence. Although there was a small magnitude of adverse difference, no strong background to prove irreversible peripheral nerve damage. Further studies with long term follow up are required to address these issues.
